# Acceptability and perceived barriers and facilitators to creating a national research register to enable ’direct to patient’ enrolment into research: the Scottish Health Research Register (SHARE)

**DOI:** 10.1186/1472-6963-13-422

**Published:** 2013-10-18

**Authors:** Aileen Grant, Jenny Ure, Donald J Nicolson, Janet Hanley, Aziz Sheikh, Brian McKinstry, Frank Sullivan

**Affiliations:** 1 Population Health Sciences, Medical Research Institute, University of Dundee, The Mackenzie Building, KirstySemple Way, Dundee DD2 4BF, Scotland; 2 Centre for Inflammation Research, Queens Medical Research Institute, 47 Little, France, Crescent, Edinburgh, EH16 4TJ,Scotland; 3 Centre for Health and Population Sciences, Hull York Medical School, University of Hull, Hertford Building, Cottingham Road, Hull HU6 7RX, England; 4 NHS Lothian Research and Development, Queens Medical Research Institute, 47 Little France Crescent, Edinburgh, EH16 4TJ, Scotland; 5 eHealth Research Group, Centre for Population Health Sciences, The University of Edinburgh, Medical School, Teviot Place, Edinburgh EH8 9AG, Scotland; 6 Division of General Internal Medicine and Primary Care, Brigham and Women’s Hospital/Harvard Medical School, 1620 Tremont Street, 3rd Floor, Boston, MA 02120-1613, USA

**Keywords:** Research register, Recruitment, Randomised controlled trial, Qualitative

## Abstract

**Background:**

Difficulties with recruitment pose a major, increasingly recognised challenge to the viability of research. We sought to explore whether a register of volunteers interested in research participation, with data linkage to electronic health records to identify suitable research participants, would prove acceptable to healthcare staff, patients and researchers.

**Methods:**

We undertook a qualitative study in which a maximum variation sampling approach was adopted. Focus groups and interviews were conducted with patients, general practitioners (GP), practice managers and health service researchers in two Scottish health boards. Analysis was primarily thematic to identify a range of issues and concerns for all stakeholder groups.

**Results:**

The concept of a national research register was, in general, acceptable to all stakeholder groups and was widely regarded as beneficial for research and for society. Patients, however, highlighted a number of conditions which should be met in the design of a register to expedite confidence and facilitate recruitment. They also gave their perceptions on how a register should operate and be promoted, favouring a range of media. GPs and practice managers were primarily concerned with the security and confidentiality of patient data and the impact a register may have on their workload. Researchers were supportive of the initiative seeing advantages in more rapid access to a wider pool of patients. They did raise concerns that GPs may be able to block access to personal patient data held in general practice clinical systems and that the register may not be representative of the whole population.

**Conclusions:**

This work suggests that patients, healthcare staff and researchers have a favourable view of the potential benefits of a national register to identify people who are potentially eligible and willing to participate in health related research. It has highlighted a number of issues for the developers to incorporate in the design of research registers.

## Background

Between 30-50% of all randomised controlled trials fail to recruit a sufficient number of participants [1]. In a speech on 5th December 2011, the United Kingdom’s (UK) Prime Minister proposed two main changes to the use of data collected in the National Health Service (NHS) in an attempt to address this issue [2]. Firstly, he advocated a public consultation on changing the NHS constitution to enable all patient data to be “*automatically included in clinical research*” with an opt-out for those who did not wish to participate; and secondly, a mechanism to provide researchers access to NHS records to identify and directly contact patients who might qualify for clinical trials. There is now a widely accepted realisation that the ability to conduct clinical research is threatened in the UK. Similar concerns are also being expressed in many other countries [3,4].

Other strategies to improve recruitment of participants to trials have recently been assessed in a Cochrane review, but with the exception of telephone reminders and opt-out strategies, most were found to be of limited value [5]. An alternative approach is the creation of a register of patients who have expressed a general interest to participate in research. Such an approach may entail registrants volunteering information about their health or patients giving permission for research teams to search their electronic health records (EHRs) to assess whether they met the eligibility criteria for studies. If so, they could then be informed about relevant studies and offered the chance to participate. Volunteer research registers already exist, varying in terms of disease [6,7], study type [8], and location such as the pioneering ‘*Volunteer for Vanderbilt’* system [9]. Registrants are notified electronically when they are a possible match to a proposed research project and they then make the decision regarding the release of their contact information. The Vanderbilt approach is now being adopted nationally in the United States of America (USA) as part of the new US Research Match program, which comprises a consortium of institutions under the aegis of the National Institutes of Health (NIH) [10]. These registers ensure that personal information is protected until volunteers authorize the release of their contact information to a specific study. MediGuard is another system which provides a free medication monitoring service and enables its 2.5 million registrants to be contacted directly about research for which they may be eligible [11]. In Scotland, the unique Community Health Index (CHI) has allowed deterministic record linkage of health data since the 1970s. This has enabled the development of exceptional data resources and long tradition of excellence in eHealth informatics research recognized by a leading role in the UK’s network of health eResearch centres funded by the MRC and a consortium of other funders: the Farr Institute [12]. The Diabetes Research Register in Scotland has already shown that response rates are likely to be higher, significantly faster and with fewer screen failures amongst patients who have previously considered and expressed interest in participation and whose electronic records (EHRs) are used to identify their suitability for studies, than the general population [13].

Given the increasing adoption of EHR systems in the UK, we believe there is a considerable opportunity to use the data within EHRs to identify people for research projects though the risks of doing so need to be addressed [14-16]. The proposed model was developed following national discussions with a range of key stakeholders, including the British Medical Association (BMA), research charities, patient representative groups, the pharmaceutical industry and the Scottish Government [17]. Instead of self-reported health data, we proposed using clinical data in medical records and linked information in laboratory and administrative databases across Scotland [18].

### A national research register - proposed process model

**Figure 1:**
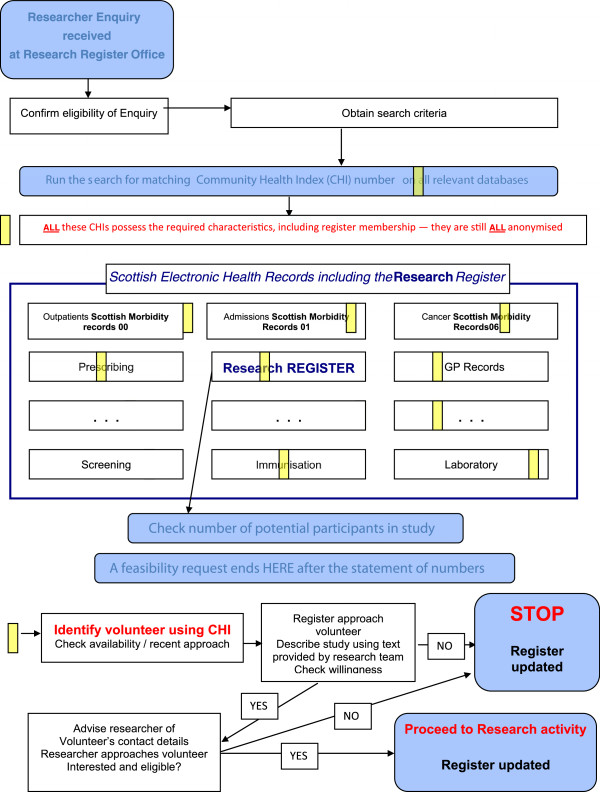
**An example of how a research study enquiry will be processed by the proposed research register.** An enquiry will be received and checked by register staff for eligibility. Once eligibility is determined search criteria will be generated and a search will be run on all relevant databases for matching Community Health Index (CHI) numbers. These databases will include the Scottish Morbidity Records (SMR), GP records and laboratory data to name a few. This search will generate a list of research interested patients with eligibility criteria for the study in question. The register staff will check if any of these people have recently been contacted about another study. If available the patient will be contacted and willingness to participate will be explored. If they say no the register will be updated and if they say yes, the researchers will be given the patients contact information and the register will be updated.

Briefly, the technology now exists to extract data from various EHRs (primary care, dental, hospital records and many more) and link this information via the patient’s CHI number. Data linkage makes it possible to identify patients who may be eligible for studies based on data held in their EHRs. The proposed register model only holds registration and contact preference data on patients; when linked to eHealth records it enables the identification of potentially eligible patients for studies Figure 1.

Patients either register via the website or completing their details on a form for secure entry by register staff. In doing so, they are providing consent for to their EHRs to be securely searched within the NHS to assess whether they met the eligibility criteria for studies which have NHS ethical and governance approval.

Researchers contact the register administrators with their inclusion and exclusion criteria, and if all the appropriate approvals were in place, an electronic search is carried out to identify eligible patients. Patients are informed by register staff about relevant studies and offered the chance to participate and if interested respond directly to the researcher.

### Aim

In the context of developing the system specification, we sought to explore the acceptability and feasibility of the national research register model to patients, clinicians, healthcare management staff and researchers to understand their perspectives on key facilitators and barriers to engagement.

## Methods

Focus groups and semi-structured interviews were conducted between February and June 2011 with patients, general practice staff from NHS Tayside and NHS Lothian and academic researchers from the local University. Focus groups were chosen for patients as the concept of a research register would be novel and focus groups present an efficient way to demonstrate new ideas, allow people to use the responses of others as a starting point, and are especially helpful in understanding nuances of attitudes, beliefs, or opinions. Interviews were considered more efficient for health service staff that have difficulty attending focus groups. This study was approved by the Tayside Committee on Medical Research Ethics B, (reference number: 11/S1402/6) and written informed consent was obtained from all participants.

### Sampling and recruitment

Research interested general practices in two Scottish health boards were identified via the Scottish Primary Care Research Network [19].

*General practices*: General practitioners (GPs) and practice managers from 10 practices were invited to take part in telephone semi-structured interviews. This was a purposive, convenience sample to gain perspectives from clinically active GPs who act as data controllers.

#### Patients

We aimed to recruit a maximum variation sample of ages, rurality, sex and deprivation, via the participating general practices. 732 patients from five practices were invited to attend focus groups by their general practice on behalf of the research team. No reminder letters were sent.

In addition, focus groups were organised with administrative members of staff from the local hospital and university to gain a wider patient perspective.

A focus group was also held with members of the local Public Patient Partnership group (PPG).

#### Research staff

Health service researchers and data analysts from the University of Dundee were invited to take part in a focus group. Participants were purposively selected with experience in data-linkage and recruitment to clinical studies.

### Data generation

The focus groups and interviews sought to establish if the proposed model was acceptable to key stakeholders and to gain an understanding of their perspectives on how the register should operate and be advertised; to inform the design, development, operationalisation and promotion of a national research register.

Prior to every focus group and interview, the facilitator gave a short presentation as to the current problems with patient recruitment to research and how a national research register was expected to work, including information about possible mechanisms for participation and data extraction and linkage mechanisms. These discussions were further facilitated by a topic guide as an aide memoire, audio-recorded and transcribed verbatim. The topic guides and presentations were modified between focus groups and interviews. Focus groups with patients and researchers lasted approximately an hour. Focus groups with patients were held in local health centres and with researchers within the University department. Telephone interviews with GPs and practice managers lasted for approximately half an hour.

### Data analysis

Focus groups and interviews were conducted by two social science researchers (AG & DN) and the focus groups were supported by a rapporteur responsible for writing notes and capturing key points. All the audio-recordings were transcribed verbatim. The transcripts were read and re-read by three of the researchers independently (DN, JU & AG). An initial coding frame was developed inductively from the interviews, and disagreements were discussed and resolved. This was then systematically applied to all transcripts. Themes were organised under related concepts derived from the topic guide and thematic analysis. The data was explored for negative cases [20,21].

## Results/findings

A total of 17 general practitioners and practice managers were interviewed, 37 people took part in seven patient focus groups and 10 health service researchers took part in another focus group.

### Patient perspectives of a national research register

The following findings are from the seven focus groups held with patients.

### Acceptability of a research register

The focus group discussions were generally positive towards the concept of a research register. Participants in the patient focus groups could understand the aims and rationale for the register and made reference to the benefits as being for the ‘common good’ and to advance research:

*“….. I mean it’s a better system than it is at the present, because you are going to get 100% response that way or near enough and the present system is that the GPs put out things on spec to people that may want to join this thing and they may get a very low return”* (Male, patient focus group 3, PPG).

And:

“*..the great advantage of a system like this is that if I was a researcher dealing with a rare disease and I wanted to get together everybody in the country, or perhaps all five of them for instance to do some trial on them, I could identify these people”* (Male, patient focus group 2).

There was a willingness to participate in the register, with the vast majority of the patient participants indicating they would be likely to join the national research register, in principle:

*“Well I think it’s a good thing to get involved in (referring to a national research register), I just felt that I wanted to do it and…quite happy….to see what happens now, as you say I can withdraw if I’m unhappy, if I’m happy I’ll just stay in….”* (Female, patient focus group 4).

And:

*“…research register seems like a great idea, why has nobody done it before?”* (Female, patient focus group 6)

The remaining participants felt they required more information about how the research register would operate, including recruitment and management processes (these issues are addressed below).

*“…I’m here to find out a little bit more about it, I don’t know enough at the moment. And I also think pre-authorising people to access my data might be an issue. I don’t know what sort of studies you’re doing”* (Male, patient focus group 3, PPG).

Although most patients perceived the register to be for the ‘common good’, those who said they were likely to register indicated that their rationale for registering would be primarily personal with the hope of future health gains for themselves or their family, so would be interested in contributing to research in areas of concern to them.

“*…I’ve got arthritis in my spine and if there’s something new or something that can help wi the pain I have, which is not constant then I’d be willing to try anything cause when it’s bad, it’s bad.”* (Female, patient focus group 1.).

Some participants did provide a more altruistic rationale for registering.

“*…I’ve been, had quite a few things done under the NHS the past two or three years so I’m quite happy to put things back”* (Male, patient focus group 3, PPG).

Our data show that the concept of a research register is acceptable to patients who volunteer for research via invitation from their GP practice. We do not suggest that all patient groups would be likely to enroll with a research register but these findings suggest that a research register is likely to recruit patients who wish to give a ‘gift’ to society or who wish to contribute for personal gain. Doubts have been expressed about the ability of biobanks to deliver on ‘promises’ [22]. It is not the intention of a national research register to make any claims about enrolment and participation in research leading to improvements in health care prevention, diagnosis or treatment regimens which may give patients false hope.

### What would expedite confidence to facilitate recruitment to a national research register?

The participants of the patient focus groups identified a number of conditions which they felt should be met in the design of a national research register and website to encourage confidence and facilitate recruitment:

#### Information about the kind of personal data extracted from patient records

Some participants wanted more information about the nature of personal data which would be extracted from patient records. They had reservations about more sensitive information being accessed as the quote below illustrates:

*“Yeah and what would be, the factual information that goes into this Register, you know in terms of actually the factual information that goes there, for instance thinking about mental health and thinking about, you know things, or disclosure or sexual abuse or, all these kind of things …”* (Female, patient focus group 4).

Some focus group participants expressed the view that that people might not wish to participate in studies about sensitive issues. Designers of the research register also considered that people may not be aware or may have forgotten their health care record contains sensitive information and access to certain information could make registrants vulnerable. Based on these observations sensitive information would not be accessed by researchers without explicit patient consent. On occasions where researchers might want access to information such as mental or sexual health they would only be provided with anonymised data within the strict ethics and governance rules and regulations which are in place to protect vulnerable patients in accordance with the proportionate governance arrangements of the Scottish Health Informatics Programme (SHIP) [23].

#### Concerns about data security

All participants wanted reassurance their data was to be handled securely. Participants recruited via the local University and NHS hospital were already informed of some of the governance and legislative issues involved in handling confidential patient data.

*“… if you don’t think your data’s secure then it's really, really going to, to put a lot of people off, you know, it just takes one slip up in that department and that’s just going to completely put people off from signing up”* (Female, patient focus group 6).

And

*“..reassurance…it would just be few hands and eyes … so it's not being passed around”* (Male, patient focus group 6).

Whereas, participants recruited via general practices assumed the electronic transfer information within the NHS was already taking place and some assumed politicians had access:

“…*I don’t mind where my data goes…I just wondered when and where and who was doing this. Was it Scottish Government or the NHS and where do your records sit? Because I would like my records to be available throughout the country, I don’t care once they’re on the internet and a doctor here can call them up, but I’m totally surprised how if you move from one practice to another your records can’t now electronically move easily.”* (Male, patient focus group 1)

And

*“Does Alex Salmond’s (Scotland’s first minister) department and does Shona Robinson from Dundee, as health minister in Scotland, are they not already looking at these figures and data?”*(Male, patient focus group 2)

All participants were informed of the research ethics, governance and legal frameworks within which research teams and the operators of the research register are accountable. Patients were reassured by this and felt this information should be made available on the register website, along with how particular studies were approved or included in the register:

”*…I understand, I understand, I am a lot more enlightened”,* (male)

“*That information should be made available for people”* (female) (Patient focus group 2).

And

*“One of the things I’d like to see is that there was a sign off on adequate training on all the legislative requirements for holding people’s data…you know if you’re going to minimise risk then everybody has to know absolutely where the legislation lies..”* (Female, patient focus group 1).

Although participants were concerned about the sensitivity of some data and the security of all data and were reassured by governance and legislative controls it was difficult to ascertain their views about these issues in relation to data extraction and linkage. Specific concerns were not raised and questions were not clearly responded too.

#### Reassurance about levels of commitment involved in the register

Despite understanding the rationale behind the register and being willing to register participants were ambivalent about making open-ended commitments. Some participants argued that registrants may not always be able to contribute to research or only to studies with minimal involvement.

*“I suppose I don’t yet understand how much of my time, what people would be asking me - would it be lots of projects?”* (Female, patient focus group 6)

And

*“Yeah, I suppose it just depends what’s like going on in your life at the time, you know how busy you are with other things.”* (Female, patient focus group 5)

Or

*“…probably ones that take up the least of your time, 'cause if you've to keep going to the surgery or go somewhere to do things, if you get a questionnaire in the post, I'd quite happily fill that in and post it back”* (Female, patient focus group 1)

During the focus group discussions participants were asked how often they thought registrants should be contacted to participate in research studies.

*“..I don’t know perhaps if I was being asked to do something every week I might think that’s a bit much, but if it was may be once every six months I would think that was alright, once a year, that’s alright…it just depends…* (Female, patient focus group 3, PPG)

There was no consensus about frequency of contact because the research studies which will hopefully utilise a research register will have differing levels of involvement. Participants felt reassured that they could have options to refuse to participate in a study which would be easy to complete and not time consuming and opportunities to withdraw if their circumstances change.

*“I mean sometimes you go into these things thinking that’s fine….it’s only an hour a month or whatever but sometimes life doesn’t give you that opportunity”* (Female, patient focus group 2).

Of more concern was that participation in one study may prevent them from taking part in another:

“…*if you’re taking part in a longitudinal study, as I have done in the past, and it’s just a question of, it’s just a kind of questionnaire about my diet and that kind of thing, would that prevent me from taking part in something else?”* (Female, patient focus group 3)

This raises questions about how participation in a national register may be ethically monitored. Some studies may involve participation in a ‘one-off’ interview or questionnaire study whereas other studies may require more intensive participation. Suggestions by researchers designing the register to resolve this issue have centred on a maximum of three invitations per year.

This section has raised some important concerns around sensitive information, data security and levels of involvement. These concerns raise the issue of who should be in control of a national research register.

### Patient perceptions of how a national research register should operate

The focus groups also set out to understand patient perceptions of how a national research register should operate, exploring issues such as ownership and control of the registers and the involvement of pharmaceutical companies.

#### Who should host a national research register?

All participants placed trust in Universities and the NHS as potential organisations to host a national research register, indicating they would prefer public ownership.

*“…but government, Universities, charities, I would’nae have a problem if they were behind it but I think the drug companies, I would step back from that”* (Female, patient focus group 6).

Within these public organisations patients would prefer access to their data to be controlled:

*“I would like, like as little people as possible, you know I don’t want every NHS staff to be able to access it, I would want core (national register proposed name) registered people, you know not even researcher, I wouldn’t want them to be able to access it, I would want them to be able to go to somebody who was in charge of the database to be able to give them the information out, I wouldn’t want a lot.”* (Female, patient focus group 5)

It was important for the patient focus group participants that a national research register was to be publically owned and controlled by public servants. One of the main reasons these participants gave for an intention to register was for the common good, so they perceived the register to be a public service and that benefits should be publically based.

#### Access to a national research register

Discussions about the register primarily focused on the implicit assumption that access would be by medical personnel and academics and research scientists employed by Universities. Participants had particular concerns about access by insurance companies largely because it was felt they could use this information to refuse to pay out on insurance claims.

*“I’m not sure if anyone’s investigated the potential implications of anyone with a life insurance policy joining something like this and whether that voids, invalidates or causes any effect on their insurance given that they can be particularly funny when it comes to claims later in life…”* (Male, patient focus group 4)

Participants had polarised views towards the potential involvement of pharmaceutical companies. They perceived involvement to be in the funding of the register or in the funding of particular studies recruiting through the register. The majority felt the involvement of pharmaceutical companies in the infrastructure of the register would put them off registering whereas others felt the involvement of these companies would help investment.

“*I can see how that would attract the pharmaceutical companies to invest (in the register) if there was this spin off effect….an economic benefit”* (Male, patient focus group 2).

And

*“I would be slightly more concerned if they went out wider commercially, say Glaxo-Klein or whatever got data as part of the research register”* (Male, patient focus group 1). Concern was focused on how ethical pharmaceutical companies would be *“…drugs company… they always have a vested interest in the outcome don’t they really?”* (Female, patient focus group 3, PPG)

For some involvement of pharmaceutical companies in finding research was a necessary evil: “…*the drug companies are just trying to make money, and yes of course they are, it’s all about money in the end of the day but if they don’t find the research for some of these the less interesting or less topical things then they, there will not be research into those things…we need to get funding from drug companies anyway, if they’re the ones with the money.”* (Female, patient focus group 3, PPG)

The involvement of pharmaceutical companies was controversial and investment by pharmaceutical companies in the infrastructure of the register is likely to influence registration. A large part of making wider health benefits a reality, such as those desired by the focus group participants (improvements in prevention, diagnosis, and treatment), will require the funding of pharmaceutical companies. This concern has ensured that when patients are invited to participate in a research study they will be informed of the funding body. This is likely to result in the register requiring a larger number of registrants for commercial than publically funded studies.

#### The promotion of a national research register

There was a general feeling across all the focus groups that a national research register would require both local and national promotion as a public good. They perceived that the register’s success would depend upon public awareness, and on highlighting the benefits of taking part in research and the contribution to society. In the general practice recruited focus groups this topic was returned to more than once.

*“…personally I think this register should be sold as the benefit to all of us from birth to death…but I believe that if you start it now you have to catch the attention of the young kids to have this evolve through 50 years…”* (Male, patient focus group 2).

They favoured promotion by a range of media:

*“Well it depends if I had the budget or if I had other mechanisms, if budget was no object the television’s obviously a great mechanism”* (Female, patient focus group 4).

And

*“…it would be something that could be advertised to everyone, like say on the television or you would hear it on the radio so you know that it’s not something, you know secretive about, a secret project, it’s about something that everybody could join in.”* (Female, patient focus group 5)

Or

*“I guess information leaflets through you know the door I guess, you know that would get to the (health board named) population…I’m not so keen on emails but certainly something through the mail is always good or, you know posters up around the hospital or in the GP surgery…”* (Female, patient focus group 6)

There was consensus among all participants, if patients were to be written to and invited to participate in the research register this should be by their general practitioner (GP):

*“I do think it (a letter) would be better coming from their GP”* (female)

*“GP, Yeah”* (female)

*“…I think in terms of response people would prefer their GP because it’s someone that they know, trust in….you’d just treat it like every other thing and probably just discard it.” (female)* (Patient focus group 1)

There was however a lack of agreement amongst patient focus group participants as to the best way to promote a national research register. Some participants were strongly in favour of invitations coming from their GP. As recruitment to research via general practices is increasingly becoming more difficult with the additional pressures on GP time, these findings suggest a range of media would be appropriate to appeal to cross sections of society.

#### Invitations to join research study

Participants were asked how they thought registrants should be invited to participate in research studies and there was agreement invitations should initially come from the research register:

“*Well, I think if initially you’ve signed up to this through a letter from your GP….I, to get the invitation for the study, that’s when I think it should come from SHARE (research register)”*( Female, patient focus group 6).

And

*“That would be fine for me, cause I know that I’ve signed up for that so I would, you know, you would expect something to come through at some point”* (Male, patient focus group 2).

There was a real sense in the patient focus groups that a national research register should be perceived and promoted as a public good, which should be in public ownership and operated by public servants. The involvement of pharmaceutical companies was contentious, with some participants perceiving their involvement in funding research studies as a ‘necessary evil’. The appeal of the register as a public good was thought to be important for inclusion in any advertising, highlighting registration as contributing to the good of society.

### Perspective of practice managers and general practitioners of a national research register

The general practitioners and practice managers interviewed were generally in favour of a national research register; they however expressed concerns over the security and confidentiality of personal patient data and the impact a register would have on their workload.

#### Acceptability of a research register

All GPs and practice managers interviewed could understand the rationale for a national research register, were aware of recruitment issues and were in favour of a research register, in principle.

*“..in principle it sounds like a good idea, I mean obviously research is very important and it's difficult to get people for research.”* (*Female GP interview 5*)

#### Security and confidentiality of patient data

Of primary concern to all GPs and some practice managers was the security and confidentiality of patient data within a national research register.

*“I think being reassured about the security of the patient data would be sort of, you know 1A on the, on the list of things that we need to, to be absolutely 100% eh reassured by before we could … agree to getting involved.”* (Male GP interview 6)

And

*“I mean from our point of view confidentiality is one of the kind of absolute … you need to be so careful about if you're giving letters about patients, there's no third party references and secure e-mails and all that - so you're thinking “where do we stand with the law?” … I, I guess you'd have to set it up in such a way that the patient was fully aware of what they were signing up to including what data would be accessed and how it would be accessed and with whom it would be shared.”* (Female GP Interview 1)

And

*“I'm not an IT expert but it just feels like the more people there are who have access potentially to patients’ records, the more chance there is that there’ll be, it, confidential information leaked and I mean I know there was an episode fairly recently in Fife where somebody was accessing the Emergency Care Summary that wasn’t supposed to be, and you know there's always that potential”* (Female GP interview 5)

As the Caldicott Guardians of all patient data held in the GP electronic patient record, it is perhaps not surprising that GPs were concerned with data security and confidentiality. Caldicott guardians are responsible for the safe use and handling of identifiable patient information. This suggests GPs will require assurances about the security of the register, including accountability and transparency mechanisms.

The first two quotes illustrate the range of views held by GPs regarding ownership and consent for use of primary care data. Views were polarised with the first quote illustrating that some GPs perceive they would have veto as to whether patient’s data was extracted and the second quote illustrates that GPs perceived consent was entirely at the patient’s discretion. This raises interesting questions for the research register, such as “*who is the custodian of the data?*” and “*can GPs veto access to patients’ records once a patient has registered?*”

#### Impact on workload

The impact on workload was a key concern for the majority of GPs and practice managers interviewed. They had two primary concerns in this regard, firstly the impact of patient queries about register or research studies, in particular, if letters were to be sent to patients on practice headed note paper. Secondly, GPs were concerned they would be involved in screening their patients to ensure they are eligible to take part in studies. Currently in the UK, GPs screen lists of patients who meet research study eligibility criteria. Despite being concerned about workload from screening patients they saw this as a necessary evil, to protect both patients and researchers.

*“…workload attached with people contacting the surgery for advice on “should I get involved in this research project or not?”…but yeah potentially that’s used up a GP appointment to do that, and that’s, that seems a bit of a, a lost opportunity… 'cause yeah, a patient may be inappropriate for one study but appropriate for another study, the same patient and not having a feel for that because often we're obviously more aware of what’s happened in patients sort of personally circumstances month to month and you know a patient that was suitable for a research projects last week may not be this week if they're, you know just had their partner, em has died or eh, something else going on their life or some other new medical condition coming along,*” (Male GP interview 6)

And

*“… I mean the work of screening that list, I guess that in itself might be a considerable job..”* (Female GP, interview 5)

Practice managers were concerned about the need to ensure that information in patient records was up-to-date.

*“…the telephone numbers of the patients constantly change and it's really even hard for us to keep updated with their telephone numbers 'cause they change their mobiles so often…. we also have a high turnover of patients as well, so you might find that by the time you get in touch with somebody they’ve left or moved..”* (Female practice manager 4)

These findings raise important issues for a national research register as to who has veto over the use of primary care data and whether GPs will be required to screen lists of patients for eligibility. The register will rely on primary care data to provide up-to-date contact information and to provide longitudinal data on patients as these are the only health care records to hold information on a patient from cradle to grave. We expect GPs to consider whether they agree to the use of the data held in their records on a study by study basis taking into account the patients’ expressed wishes to be contacted for studies in which they are potentially eligible. These findings show GPs are an important group of people to have on board with the register and promotion of the research may need to extend beyond patients.

### Health service researchers’ perspectives of a national research register

The following findings are from one focus group with 10 health service researchers, data analysts and an IT manager from across primary and secondary care based research.

#### Benefit of a research register

Researchers were supportive of the initiative seeing a national research register as having the potential to overcome their problem of accessing and recruiting patients and offering the advantage of more rapid access to a wider pool of patients.

*“..you can only contact them through their GPs, for specific studies, whereas this would be a much wider opportunity.”* (Male clinical researcher, research focus group)

And

*“Well, I’m sure the benefits for patients is more that you’re giving them more opportunities to take part in research. There are a group of patients who object to the GP being the gate-keeper and feel that they should be asked rather than the GP deciding whether they should be asked is the main benefit”* (Female health services researcher, research focus group).

Although supportive of a national research register, researchers raised some issues they felt needed to be addressed to ensure the functionality of a register:

#### Concern a national register may not be representative

Some researchers raised concerns GPs may be able to block patient involvement in the register by refusing access to patient’s personal data or by not giving permission for the data-extraction to take place from their clinical system:

*“It has to be through the GP so if their GP doesn’t want to be involved, you can be losing a great amount of patients that may be willing to take part in this research and it is because their GP is gatekeeping”* (Female health service researcher, research focus group).

And

*“…you will have a two-tier system whereby you would have presumably a very large cohort of patients who had agreed and whose GPs had agreed to make the data available and then perhaps a much smaller sub-group of people who had agreed and for one reason or another the GPs felt they didn’t want to share the data..”* (Male clinical researcher, research focus group).

This also raised the issue about whether GPs can veto patients’ participation in the research register.

Others were concerned that only the middle classes would register:

*“Is there a danger…that you’re only going to get these very middle class people so all the trials end up being done on people who have not really got anything wrong with them?”* (Male health services researcher, research focus group)

This is a common problem in research, in particular with studies which use opt-in models. An important issue for some studies hoping to use the register will be the representativeness of registrants. It is therefore intended that targeted advertising and social networking will attract patients from a wide range of groups in society.

#### Measures are required to ensure eligibility of patients

Researchers expressed concern about contacting deceased patients and would like to see some measures in place to protect against this in the national research register:

*“I take it one of the benefits we’re looking for is to prevent what’s happened in the past, people making contact with patients who have been deceased since and the accuracy…..what’s the lag between the register being updated and someone actually being approached. There’s examples over the last fifteen months were patients were approached through various registers and they’d died.”* (Male data analyst, research focus group)

And

*“If someone has said I’m happy to be contacted and some researcher contacts him unfortunately after he’s died, it’s not acceptable….you need to make sure that you didn’t do it again”* (Male health services researcher, research focus group).

And

*“That comes back to the idea that maybe this does need….at least the request to participate does need to be routed via the GP, even if it’s electronically to establish that they’re still alive and what conditions they have and then you approached them”* (Male health services researcher, research focus group).

GPs record when a patient is deceased and the register can also be linked to the Scottish General Registrar Office (GRO), the research register will extract data from GP practice systems nightly so the information used by the research register will be the most up-to-date and the chances of a deceased patient being identified are minimal. There are other reasons for which GPs screen patient lists, such as a close family member recently passing away.

## Discussion

There was clear support for the concept of a research register to facilitate recruitment to research studies from patients, practitioners and researchers. Although supportive, some patients and GPs require assurances about data security and accountability. Without these assurances in place researchers were concerned the register may not be representative and/or GPs may be able to veto patient’s participation in the register.

Scotland has a public sector health service covering the entire population, and using a single patient identifier for all health records. This offers a unique opportunity, with cradle to grave records on the whole population in a range of EHRs (primary care, hospital, dental, prescribing, laboratory, screening and immunisation records). There was general agreement that these records should be used for the public good, an assumption by patients that at some levels this was already being done, and no questions were asked about the technical ability to data link, extract or search the records of those on the register.

For the patient groups in our study the motivation for participation in a national research register was largely self-interest although some were happy to volunteer without any expectation of direct benefit to them. Any promotion of a national register must be careful not to give patients false hope and to promote participant altruism. For those who chose to give a ‘gift’ to society it is important to ensure their data appropriately cared for, ensuring data confidentiality and security.

Patients would like to see a public body, operated by public servants in control of a national research register. This implies high levels of institutional trust for these organisations [24]. Their lack of questioning of the data linkage and extraction technology can be viewed as implied trust of NHS staff, researchers and the NHS IT infrastructure. Although, some participants sought reassurance about how secure and confidential their personal data would be implying levels of conditional trust. However, lack of trust or conditional trust does not mean distrust [25]. Unsurprisingly, there were differing levels of trust, highlighting for some members of society, trust in the register will not be voluntarily given and accountability information, reassurance and evidence of successful operations will be required. For those groups who give their trust this should be highly valued.

Patients favoured an approach which focuses on the social good and public nature of this research, leading participants, by a majority, to reject the notion of investment by pharmaceutical companies in the register infrastructure. Whereas views were polarised over use of the register for commercial gain by making it available to research studies funded by pharmaceutical companies. This issue may have been different in other healthcare systems where private providers predominate. However, the issue of ownership and control of personal data when it is to be exploited for commercial gain has arisen in many different healthcare systems [26,27]. Legal decisions on this vary, but for a voluntary register based on trust to be successful, complete transparency will be required. When current ethical and legal restrictions were described to patients in our study this seemed to reassure people, however more detailed exploration of what is likely to be acceptable to the wider Scottish public is required. The development of a national register needs to be within an accepted legal, ethical and governance framework.

As the Caldicott Guardians and custodians of patient’s cradle-to-grave health record it is advantageous if a national research register has the backing of general practitioners. Researchers participating in this study were concerned GPs would be able to veto a patient’s consent to take part in the register. GPs are not in a position to veto patients consent to take part in a national research register, but GPs have total control over access to data held in their clinical systems, and will be able, to some extent, to influence volunteer uptake. It is likely GPs will be able to influence uptake or access to data at three stages of the research register process: 1) registration; 2) data access; and 3) utilisation. As a result it is important to address the concerns raised by GPs about the impact on their workload and provide reassurances regarding accountability and market a national research register to health professionals as well as patients.

Patients desired a range of media to promote a national register, emphasising this is a venture which is not suited to a ‘one size fits all’ approach. Given the diversity of research studies which are likely to use the research register and levels of contribution required from each, the register must be able to attract a number of registrants from different societal groups to be able to offer a comprehensive service, which caters to the needs of all research studies.

### Strengths and limitations of the study

These findings have highlighted a number of important issues for the key stakeholders in a national research register. A strength of this research is its timing, allowing these findings to inform the design and development of a national research register and contribute to a wider debate and national programme of research and development of EHRs [28].

This study was only conducted within two health boards in Scotland to gain an understanding if a research register was acceptable to patients, health professionals and researchers. Unfortunately, we had a low response rate to invitations to participate in the patient focus groups. This may be a reflection of a lack of interest or may be due to the fact no reminder letters were sent and patients were only invited once. The views of policy commissioners were not explored. As in any study, we had to make choices in our study design to balance ideal recruitment figures with feasibility and resource constraints. Only one focus group was held with health services researchers and those recruited had extensive experience in data-linkage and large database studies. As a result their views may not be representative of all health services researchers working with a range of methodologies. Likewise, the GPs and practice managers had previously taken part in research and their views may not be representative of their professional groups. Although two thirds of Scottish general practices are research active (Alison Hinds, Scottish Primary Care Research Network, personal communication). There was clear enthusiasm among the patients we spoke to, but these people were to some extent self-selecting and younger people in particular were under-represented. Previous research has suggested that younger people are concerned about allowing access to their personal information [29]. It is not clear if the general public would be so altruistic; many of these people were recruited via general practice and may place high levels of trust within the NHS and public institutions. However, we believe seventeen interviews with professionals, seven focus groups with patients and one focus group with health services researchers provided a sufficient level of evidence for an explorative study of this nature.

### Comparison with other studies

Although a national research register is a novel concept the acceptability of disease specific registries has been shown [6,7,30]. The acceptability and knowledge of ethical and legal governance regulations was important, concern about privacy, security and consent could stifle a national research register [31,32] and studies which seek representative samples [33]. As others have found, patients had concerns about the frequency of requests to participate, the extent of commitment required, and who the sponsors potentially may be within disease specific registers [33]. Patients placed high levels of trust in GPs and the NHS, confirming other studies [34]. For patients, their trust was tempered by a concern to know more about the practicalities of the process. Patients and health practitioners wanted greater clarity on nature of recruitment and data management processes as a basis for this trust, mirroring the findings of others [30,35,36].

Recruitment to clinical studies is a major concern for all healthcare researchers [5]. There is also evidence that many people would like to participate in medical research if they knew of suitable projects. This has been demonstrated in the United States by Mediguard and Research Match [10,11].

#### Implications for future research

These findings have raised a number of important design and operationalization issues but also highlighted issues which will require further public engagement.

This study did not explore the views of patients towards the secondary use of their data. Further research is required to establish how patients perceive their consent to a national research register and associated research studies. Establishing patient views towards the secondary use of their personal data was beyond the scope of this study however, the concern by patients about the security and confidentiality of their data raises this as an issue for further consideration [37].

Participants in the patient focus groups rarely mentioned concerns about sensitive data such as sexual or mental health unless probed. Researchers were concerned about contacting deceased patients. More detailed exploration about the handling of sensitive information within a national research register is required.

Since this research study was completed a ministerial announcement has been made by the Scottish Health Minister that the register has been adopted by NHS Research Scotland (NRS) and branded SHARE (Scottish Health Research Register) [18,38]. The national researcher register will operate through the existing infrastructure and operating procedures of the Scottish Health Informatics Programme [18]. There has been extensive development and pilot work carried out by the Health Informatics Centre at the University of Dundee since the completion of this work and the SHARE process has been influenced by these findings.

## Conclusions

Providing suitable safeguards are in place, patients, health service staff and researchers have a favourable view of the potential benefits of a national register held within the public sector to identify people who are potentially eligible and willing to participate in health related research. UK Government policies are encouraging greater use of the data in EHRs to encourage more rapid progress with clinical research, though consent cannot be assumed [39,40]. The Nuffield Council on Bioethics Working Group has recommended that the patients’ right to opt-out of a database system at any time should be recognised; that the patients should be asked explicitly to consent to the sharing of their records outside of the primary care environment; and that organisations responsible for sharing EHRs engage more effectively with patients to identify what forms of data sharing are accepted as legitimate [41].

## Abbreviations

GP: General practitioner; NHS: National health service; EHR: Electronic health record; NIH: National institute for health; UK: United Kingdom; USA: United States of America; BMA: British medical association; IT: Information technology; NRS: National research Scotland; SHARE: Scottish health research register.

## Competing interests

The authors have an interest in encouraging more effective and efficient recruitment to clinical trials and other methodologically rigorous research. They have no direct financial interest in the existence of a research register.

## Authors’ contributions

FS conceived and AG designed the study. AG, DN and JU analysed the data. AG and FS drafted the manuscript and AG is guarantor. All authors helped interpret the results and commented on drafts of the manuscript. All authors read and approved the final manuscript.

## Pre-publication history

The pre-publication history for this paper can be accessed here:

http://www.biomedcentral.com/1472-6963/13/422/prepub
